# A Novel Detection Platform for Shrimp White Spot Syndrome Virus Using an ICP11-Dependent Immunomagnetic Reduction (IMR) Assay

**DOI:** 10.1371/journal.pone.0138207

**Published:** 2015-09-18

**Authors:** Bing-Hsien Liu, Yu-Chen Lin, Chia-Shin Ho, Che-Chuan Yang, Yun-Tsui Chang, Jui-Feng Chang, Chun-Yuan Li, Cheng-Shun Cheng, Jiun-Yan Huang, Yen-Fu Lee, Ming-Hung Hsu, Feng-Chun Lin, Hao-Ching Wang, Chu-Fang Lo, Shieh-Yueh Yang, Han-Ching Wang

**Affiliations:** 1 MagQu Co., Ltd., New Taipei City 231, Taiwan; 2 Institute of Biotechnology, National Cheng Kung University, Tainan 701, Taiwan; 3 Institute of Bioinformatics and Biosignal Transduction, National Cheng Kung University, Tainan 701, Taiwan; 4 Graduate Institute of Translational Medicine, College of Medical Science and Technology, Taipei Medical University, Taipei 110, Taiwan; Uppsala University, SWEDEN

## Abstract

Shrimp white spot disease (WSD), which is caused by white spot syndrome virus (WSSV), is one of the world’s most serious shrimp diseases. Our objective in this study was to use an immunomagnetic reduction (IMR) assay to develop a highly sensitive, automatic WSSV detection platform targeted against ICP11 (the most highly expressed WSSV protein). After characterizing the magnetic reagents (Fe_3_O_4_ magnetic nanoparticles coated with anti ICP11), the detection limit for ICP11 protein using IMR was approximately 2 x 10^−3^ ng/ml, and the linear dynamic range of the assay was 0.1~1 x 10^6^ ng/ml. In assays of ICP11 protein in pleopod protein lysates from healthy and WSSV-infected shrimp, IMR signals were successfully detected from shrimp with low WSSV genome copy numbers. We concluded that this IMR assay targeting ICP11 has potential for detecting the WSSV.

## Introduction

White spot disease (WSD), which is caused by white spot syndrome virus (WSSV), is a globally important shrimp disease [1, 2]. Over the past 20 y, WSD has caused huge economic losses to shrimp aquaculture around the world. All life stages of penaeid shrimps, i.e. from egg to brooder, are potentially susceptible to WSSV. In shrimp hatcheries, the virus can be transmitted vertically from WSSV-positive brooder to offspring [3]. Furthermore, cannibalization of WSSV-infected, moribund shrimp is primarily responsible for horizontal transmission [4]. Therefore, effective diagnostic methods and appropriate biosecurity and management are critical to prevent the spread of WSD.

There are several systems for detecting WSSV. A high sensitivity, polymerase chain reaction (PCR) based system for WSSV is now commercially available and popular in large-scale shrimp farms [5–9]. However, the relatively high cost of this assay, plus the need for specialized equipment and technical expertise, limits its use in small-scale operations. Therefore, an immune-based detection system has appeal as an alternative, due to its lower cost and technical requirements [10]. For example, home pregnancy tests are a widely used, immune-based detection system [11]. Immune-based WSSV diagnostic systems using gold-labeled antibodies or other platforms have been developed [10, 12–15]. The sensitivity of the assay depends on the target protein. Current immune-based WSSV diagnostic systems target WSSV structural proteins, which assemble to form WSSV virion particles [16].

Before “omic” approaches were developed, specific WSSV structural proteins, e.g. VP28, VP24, VP19 and VP15, were thought to be the most highly expressed genes/proteins. Consequently, these WSSV structural proteins were the targets detected in immune-based WSSV diagnostic systems [16]. However, using transcriptomics and proteomics, Wang *et al*. [17] reported that ICP11 was the most highly expressed WSSV gene/protein. This nonstructural protein is expressed in the late stage of WSSV replication and functions as a DNA mimic protein which interferes with nucleosome structure and causes cell death [17, 18]. In addition to being expressed at a high level (compared to VP28), ICP11 is very soluble and can be easily detected in WSSV-infected tissue lysate using methods that are inexpensive and technically simple [17]. Siriwattanarat *et al* [19] subsequently developed a monoclonal antibody against ICP11 that had stronger immunoreactivity than other monoclonal antibodies specific for VP28 and VP19, indicating that this could improve detection sensitivity for WSSV. Therefore, we inferred that ICP11 was both a good indicator of WSSV infection and a suitable candidate for an immune-based diagnostic system for this pathogen.

**Fig 1:**
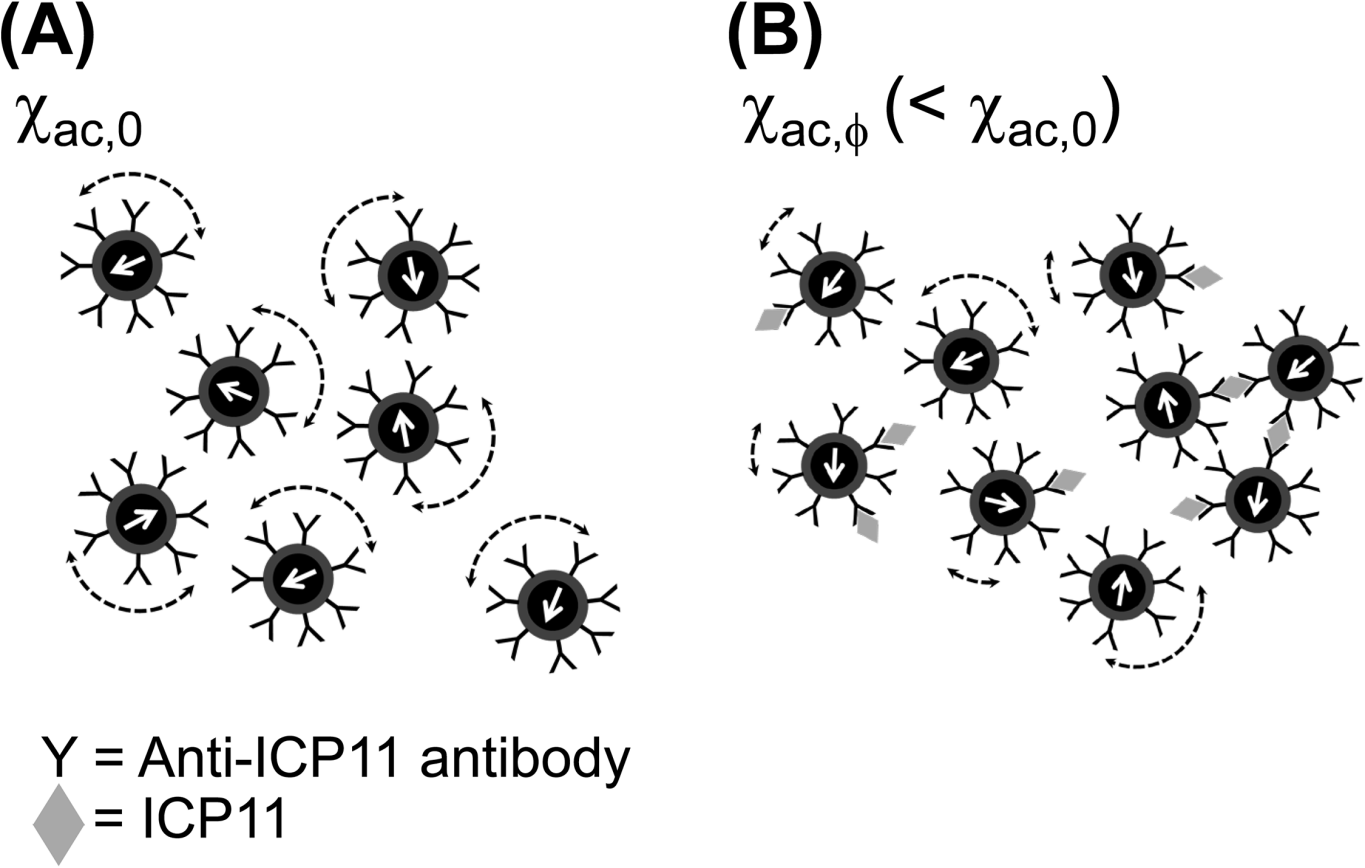
Overview of immunomagnetic reduction (IMR). (A) Each magnetic nanoparticle, bio-functionalized with antibodies against target proteins, oscillates with the applied alternating current (AC) magnetic field before binding with ICP11. χ_ac,0_: the original multiple-frequency AC magnetic susceptibility of the magnetic nanoparticles (B) When these magnetic nanoparticles bind to target proteins, they become larger, and some even form clusters. This reduces the AC magnetic susceptibility of the reagent. χ_ac,ϕ_: the resulting magnetic susceptibility of magnetic nanoparticles after binding with the target proteins.

Instead of current immune-based diagnostic systems that involve antibodies against epitopes of target protein, the immunomagnetic reduction (IMR) assay we use here provides an ultra-high sensitivity immune-based detection with a single antibody. In an IMP assay, a specific antibody that recognizes the target protein is immobilized on magnetic nanoparticles, and thus enables them to specifically bind to the target protein. Magnetic nanoparticles in reagents oscillate with alternating current (AC) magnetic fields via magnetic interaction. Therefore, under the influence of external AC magnetic fields, the original reagent containing homogeneously dispersed magnetic nanoparticles generates a magnetic signal, called multiple-frequency ac magnetic susceptibility χ_ac,0_ (Fig 1A). After target proteins are bound to antibody-labeled magnetic nanoparticles, the resulting magnetic susceptibility is designated χ_ac,ϕ_ (Fig 1B). Due to the formation of magnetic clusters, χ_ac,ϕ_ is smaller than χ_ac,0._. Thus, the reduction in χ_ac_ of the magnetic reagent is used to determine the concentration of the target protein. Due to cost efficiency, quantification and ease of automatization, the IMR platform is becoming increasingly important as a diagnostic approach. In addition to its use in human diseases [20–23], an IMR assay was also used to detect Nervous Necrosis Virus (NNV), a major viral pathogen for grouper and other fish [24–25].

The objective here was to develop a sensitive IMR-based diagnostic system for WSSV that targeted the ICP11 protein. First, an anti-WSSV ICP11 antibody was conjugated onto Fe_3_O_4_ magnetic nanoparticles; thereafter, particle diameter, time-dependent AC magnetic susceptibility and concentration-dependent IMR signals were determined. Second the, detection efficiency of the WSSV ICP11 IMR assay was determined by comparing the ICP11-IMR signals in lysates extracted from shrimp pleopods to the WSSV virion copy numbers (assessed with real-time PCR).

## Materials and Methods

### Virus, virus inoculum and experimental shrimp

The virus used was a WSSV Taiwan isolate (GenBank accession no. AF440570), with the WSSV stock prepared as described [26]. The experimental inoculum was diluted from this stock with PBS (137 mM NaCl, 2.7 mM KCl, 10 mM Na_2_HPO_4,_ and 2 mM KH_2_PO_4_).

Shrimp (*Litopenaeus vannamei*; ~10 g body weight) were purchased from a commercial market. After acclimation for 1~2 d in water tank systems containing sterilized seawater (30 ppt at 25~27°C), shrimp were divided into two groups: a PBS-injected group (control) and an infected group. Control shrimp were injected with PBS, whereas infected shrimp were challenged with WSSV inoculum (2.2 WSSV copies/μl; 100 μl/shrimp) by intramuscular injection. At 24-h post injection, pleopod samples from both groups were collected and subjected to IMR and real-time PCR.

### Determination of WSSV genome copy number in shrimp pleopod samples

To determine the infection level in WSSV-infected shrimp, we used the IQ REAL^TM^ WSSV Quantitative System (GeneReach Biotechnology Corp., Taiwan), which is an absolute quantitative method that measures the amount of viral genomic DNA. Pleopod samples were collected as described previously [27] and total genomic DNA (shrimp and viral) extracted with a DTAB/CTAB DNA extraction kit (GeneReach Biotechnology Corp., Taiwan). A real-time qPCR reaction was performed on a CFX Connect^TM^ Real-Time PCR machine (BioRad) following the IQ REAL^TM^ WSSV Quantitative System manufacturer’s instructions. The Dual P (+) Standard provided by the kit contains 10^6^ copies/μl of fragments of both WSSV and shrimp genomic DNA. This standard was series-diluted to generate a standard curve (10^6^, 10^5^, 10^4^, 10^3^, 10^2^, and 10^1^ copies/μl) which was used to determine virion copy numbers.

### Expression and purification of recombinant ICP11

The full-length ICP11 gene was cloned into a pET 21b expression vector (Novagen). Recombinant ICP11 protein fused with a C-terminal His6-tag was expressed in *E*. *coli* BL21 (DE3) cells at 20°C and purified as described previously [17, 18]. The resulting recombinant ICP11 protein was verified by SDS-PAGE.

### Extraction of protein lysate from shrimp samples for Western blot analysis

To extract the protein lysates for Western blot analysis, the pleopods from healthy and WSSV-infected shrimp were homogenized in lysis-PBS buffer (PBS diluted 3x in ddH_2_O at 4°C) with protein inhibitor and centrifuged at 10,000 xg for 15 min. The concentrations of the total protein lysates in the supernatant were then quantified using a Bio-Rad Bradford protein assay (Bio-Rad). After mixing with SDS-sample buffer (1% SDS, 15% glycerol, 10 mM Tris-HCl [pH 6.8], 10% beta-mercaptoethanol), samples (20 μg) were separated on 15% SDS-PAGE. After separation, samples were either stained with Coomassie Brilliant Blue, or else transferred onto a polyvinylidene difluoride (PVDF) membrane, incubated with anti-ICP11 antibody or anti-VP28 antibody, and then detected with a horseradish peroxidase (HRP)-conjugated secondary antibody. Detected proteins were visualized using an ECL detection system (Perkin-Elmer).

### Extraction of protein lysate from shrimp pleopod samples for the immunomagnetic reduction (IMR) assay

Total shrimp pleopod samples (15 mg per group) were homogenized in 200 μl lysis-PBS buffer at 4°C, kept on ice for 5 min, and then centrifuged (10,000 xg for 10 min) with the supernatants being collected in 1.5 ml microcentrifuge tubes. Total protein concentrations were measured with a Qubit® 2.0 fluorometer (Invitrogen), and the protein lysates were stored at -20°C until the IMR assay was performed.

Detailed illustrations for IMR have been published [22, 28]. The reagent contained homogeneously dispersed Fe_3_O_4_ magnetic nanoparticles (MF-DEX-0060, MagQu, Taiwan), which were coated with hydrophilic surfactants and a polyclonal anti-ICP11 antibody [17].

The antibody was covalently bound onto the magnetic nanoparticles as described previously [24, 29] or by using a commercial kit (KT-COO-0060, MagQu, Taiwan). The size distribution of the Fe_3_O_4_ magnetic nanoparticles coated with anti ICP11 was analyzed with dynamic laser scattering (Nanotrac 150, Microtrac). For testing, 40 μl of magnetic reagents (Fe_3_O_4_ magnetic nanoparticles coated with anti ICP11) was mixed with 60 μl total protein lysate (diluted 200x) and an immunomagnetic analyzer (XacPro-E, MagQu, Taiwan) was used to assess the IMR signals. The percent reduction in the χ_ac_ of the reagent was defined as the IMR signal, IMR (%), and was calculated as follows:

$$\text{IMR}\;\left(\%\right)=(\chi_{\text{ac},\text{o}}\;\text{-}\;\chi_{\text{ac},\phi })/\chi_{\text{ac},\text{o}}\text{x}\;100\%$$ [Formula 1]

## Results and Discussion

### Expression of WSSV ICP11 and VP28 in WSSV-infected shrimp

**Fig 2:**
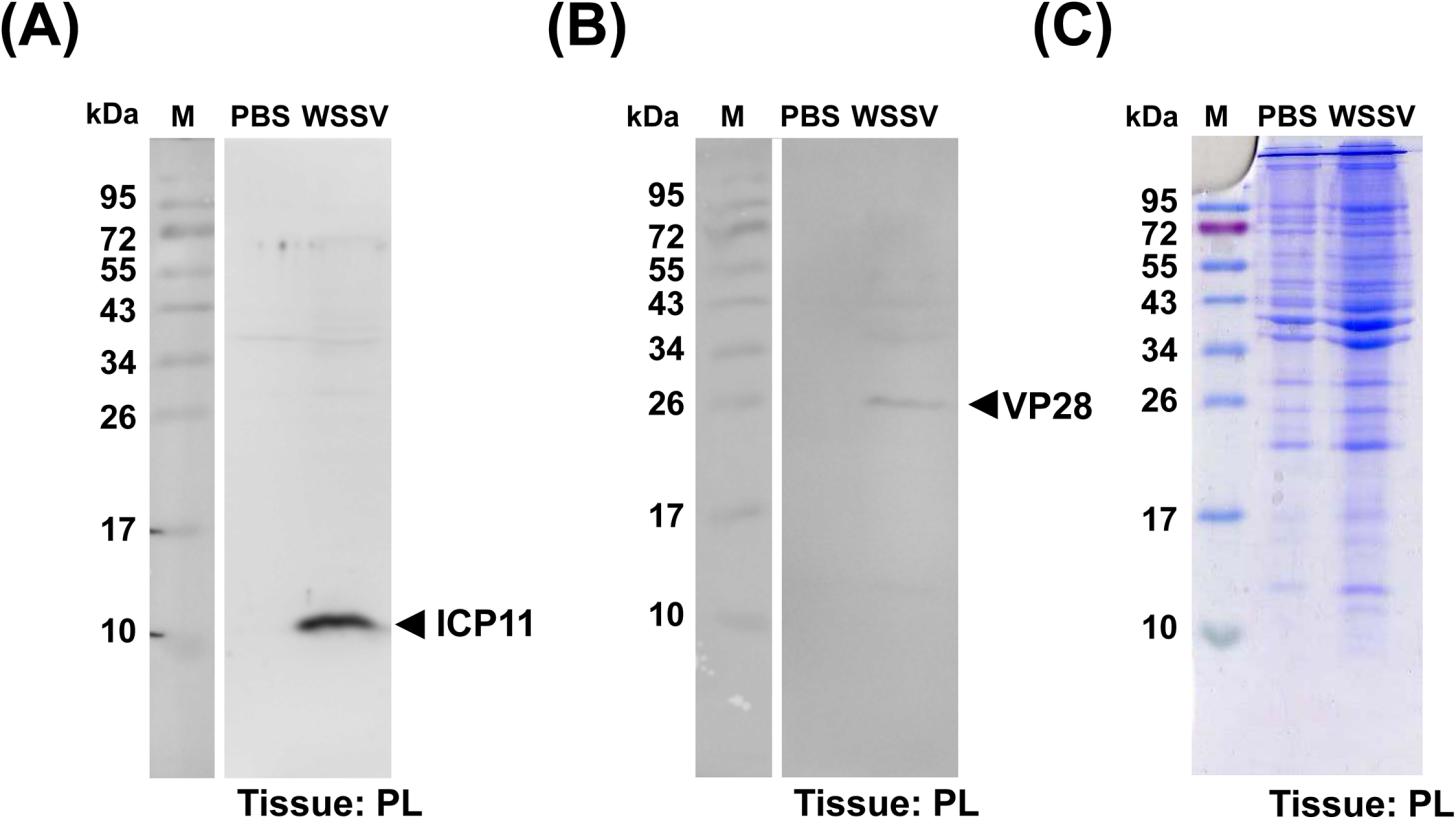
Western blot analysis of WSSV ICP11 and VP28 in total lysates from shrimp pleopods (PL) collected from healthy (PBS) and WSSV-infected shrimp. After separation and transfer to PVDF membrane, samples were probed with (A) anti-ICP11 antibody or (B) anti-VP28 antibody. (C) To show the protein loading, the SDS-PAGE was stained with coomassie blue. M: Prestained Protein Ladder (10–170 kDa)

As shown in Fig 2, a protein band of approximately 11 kDa was recognized by the anti-ICP11 polyclonal antibody only in the protein lysates from the pleopods of WSSV-infected shrimp. By contrast, Western blotting with the WSSV VP28 antibody detected a relatively faint 28 kDa band. No non-specific signals were detected by either antibody, suggesting that the anti-ICP11 antibody and anti-VP28 antibody both had a specificity against their respective targets. After this experimental demonstration of the high specificity of the anti-ICP11 antibody, we considered this polyclonal antibody against ICP11 to be suitable for the subsequent IMR assays. These results also show that, compared to VP28, the lysates contained a relatively large amount of WSSV ICP11, which should make it a good target for an immune-based detection system.

### Characteristic of the ICP11 IMR

**Fig 3:**
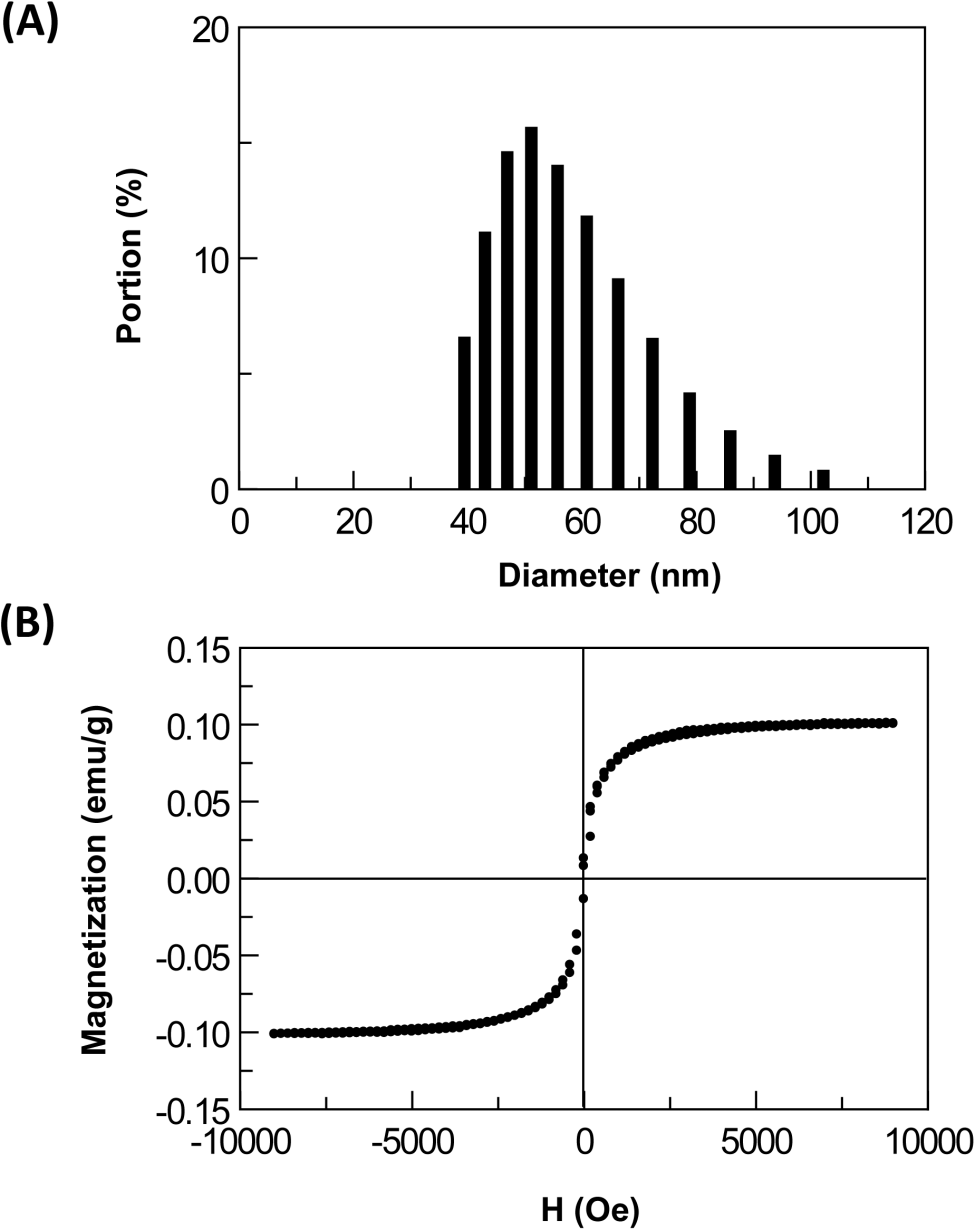
The magnetic reagent used in this study. (A) Distribution of the diameter of anti-ICP11 coated Fe_3_O_4_ nanoparticles. (B) Magnetic hysteresis curve of reagent containing anti-ICP11 coated Fe_3_O_4_ nanoparticles.

Magnetic nanoparticles were 55.4 ± 12.3 nm (mean ± SD; Fig 3A), with a saturated magnetization of 0.1 electromagnetic unit (emu)/g (Fig 3B).

**Fig 4:**
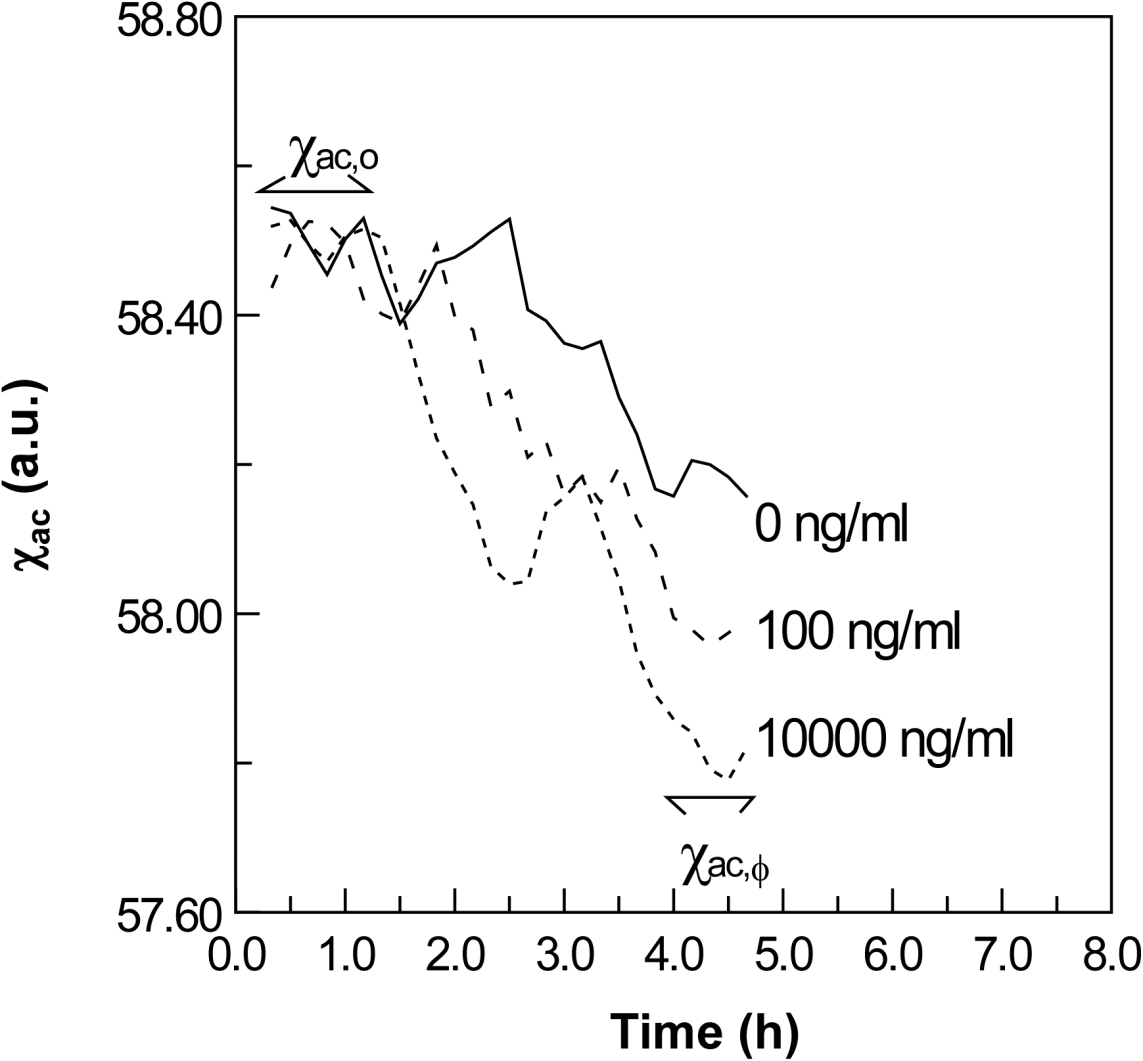
Time-dependent AC magnetic susceptibility of reagent reacting with rICP11 protein in PBS solution with various concentrations of ICP11.

To determine the immune reactivity of the magnetic reagent containing the Fe_3_O_4_ magnetic nanoparticles coated with anti ICP11, the reagent was mixed with varying concentrations of recombinant ICP11 protein (0−10^4^ ng/ml) and IMR was used to determine the time-dependent ac magnetic susceptibility. The continuous real-time magnetic response, χ_ac_, for the various concentration of ICP11 is shown in Fig 4. The signal from the magnetic reagent with 0 ng/ml ICP11 was used as a negative control. After 1.5 h, the χ_ac_ started to decrease and mostly continued to decrease through to the termination of measurement at 4.5 h.

At 4.5 h, although there was a reduction in the χ_ac_ signal in the negative control, the reduction was clearly greater in the samples with 100 or 10,000 ng/ml rICP11. Based on these reductions, we concluded that the magnetic nanoparticles reacted with the ICP11 protein molecules.

### IMR signals as a function of rICP11 concentration

**Fig 5:**
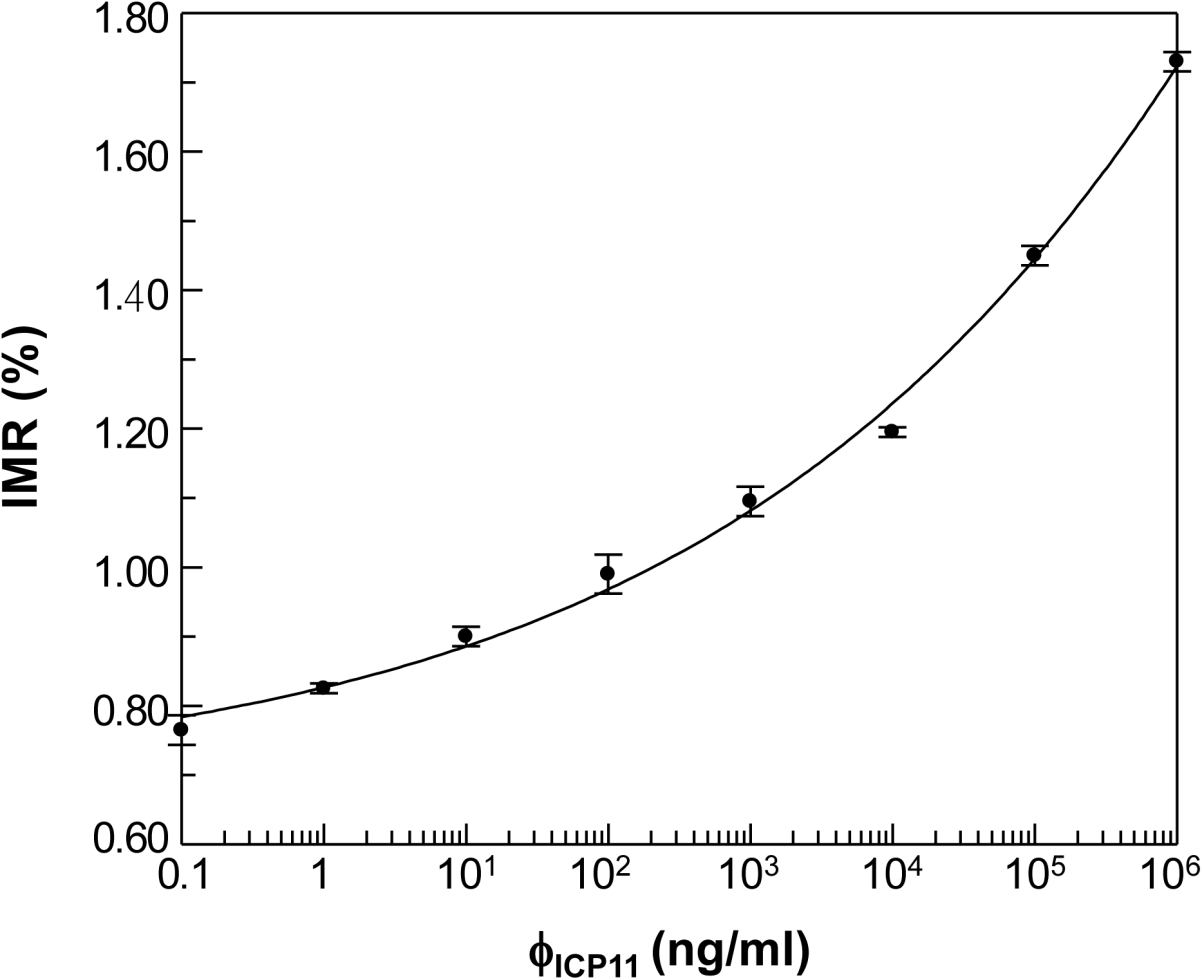
IMR (%)–ϕ_ICP11_ (spiked-rICP11-concentration in PBS) curve showing concentration-dependent IMR signals for ICP11 with standard deviations (duplicate measurements).

To establish the relationship between rICP11 concentration and the percent reduction in χ_ac_ signals from the magnetic reagent, the IMR (%) signals were plotted against rICP11 concentration (ϕ_ICP11_) (Fig 5). As the rICP11 concentration increased from 1 ng/ml to 0.1 x 10^6^ ng/ml, the corresponding IMR signal also increased. A measurement of the reliability of these IMR signals is given by the coefficient of variation (CV), which is defined as the ratio of the standard deviation to the mean value of the detected signals. Usually, no matter how strong/weak the signal is, the CV should be less than 3%, which corresponds to high precision. As shown in S1 Table, all of the CV values were below this threshold, and we therefore conclude that the detected IMR signals shown in Fig 5 are reliable. The relationship between IMR (%) and ϕ_ICP11_ can be described as follows:

$$\text{The relationship between IMR}(\%)\;\text{and ICP}11=\frac{A-B}{1+{\left(\frac{\phi_{ICP11}}{\phi_{o}}\right)}^{\gamma }}+B,$$ [Formula 2]

in which A, B, ϕ_o_, and γ are fitting parameters.

Using the data in Fig 5, the outcome was: A = 0.678, B = 9.948, ϕ_o_ = 1.04 x 10^12^, and γ = 0.15. The resulting fitting curve was plotted using a solid curve (Fig 5). The coefficient of determination (R^2^) between the data points and the curve was 0.996. It is worth noting that A in the logistic function denotes the noise level for the IMR signal (i.e. IMR signal at ϕ_ICP11_ = 0). This formula can also be used to calculate the ICP11 concentration of shrimp samples.

### Detection limit of the ICP11 IMR platform

The detection limit is defined as the lowest concentration of target protein that produces an IMR signal at least 3 standard deviations greater than the baseline noise level. The standard deviation for IMR at low concentrations (e.g. 0.1 ng/ml ICP11) was 0.02% (See S1 Table). Since the baseline noise signal, A, is 0.678, the limit of detection in terms of the IMR signal is (0.678 + 3 x 0.02) % = 0.738%. Based on Formula 2, this would give an ICP11-IMR detection limit of ~0.002 ng/ml.

### Linear dynamic range (LDR) of the quantitative ICP11 IMR

**Fig 6:**
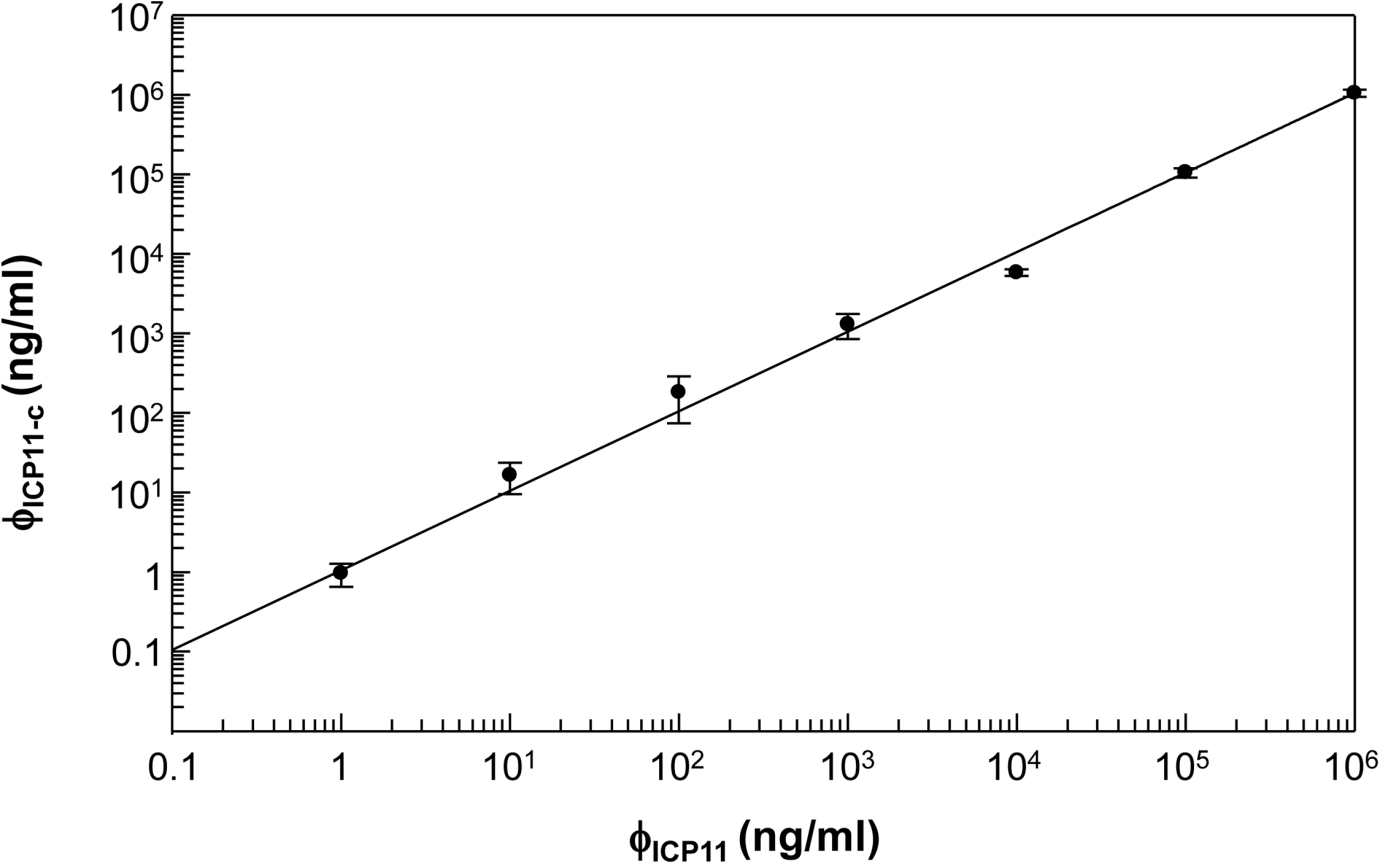
Linear correlation between actual ICP11 concentration (ϕ_ICP11_) and ICP11 concentration (ϕ_ICP11-c_) as measured by IMR in Fig 5.

To convert IMR signals into ICP11 concentration (denoted ϕ_ICP11-c_), the detected IMR signals (Fig 5) were applied to the logistic function. The relationship between ϕ_ICP11-c_ and ϕ_ICP11_ was plotted in Fig 6. The slope of the ϕ_ICP11-c-_ ϕ_ICP11_ curve was 1.05, and coefficient of determination (R^2^) was 0.999. The requirement for determining the range of linearity in terms of ICP11 protein concentration is that the slope of the ϕ_ICP11-c-_ ϕ_ICP11_ curve lies between 0.9 to 1.1. Hence, these results demonstrate that the IMR assay for ICP11 protein has a linear dynamic range from 0.1 to 1 x 10^6^ ng/ml.

### IMR assay to detect ICP11 protein in protein lysates extracted from pleopods of healthy and WSSV-infected shrimp

Pleopods were collected from both the control (PBS-injected) and WSSV-infected groups. One of the pleopods from each shrimp was used to measure the WSSV viral copy number by real-time PCR, while the other pleopod was used for the IMR assay.

Based on the IQ REAL^TM^ WSSV Quantitative System (which determines the number of WSSV copies per mg tissue), samples from the WSSV-infected group were categorized into two subgroups: light infection (7 samples; 10 ~ 3100 WSSV copies/ mg tissue) and severe infection (6 samples; 1.57 x 10^4^ ~ 2.83 x 10^5^ WSSV copies/mg tissue). Although control shrimp were not experimentally challenged with WSSV, a low level infection (0.08 ~ 7 WSSV copies/mg tissue) was detected, which we inferred was due to persistent infection.

**Fig 7:**
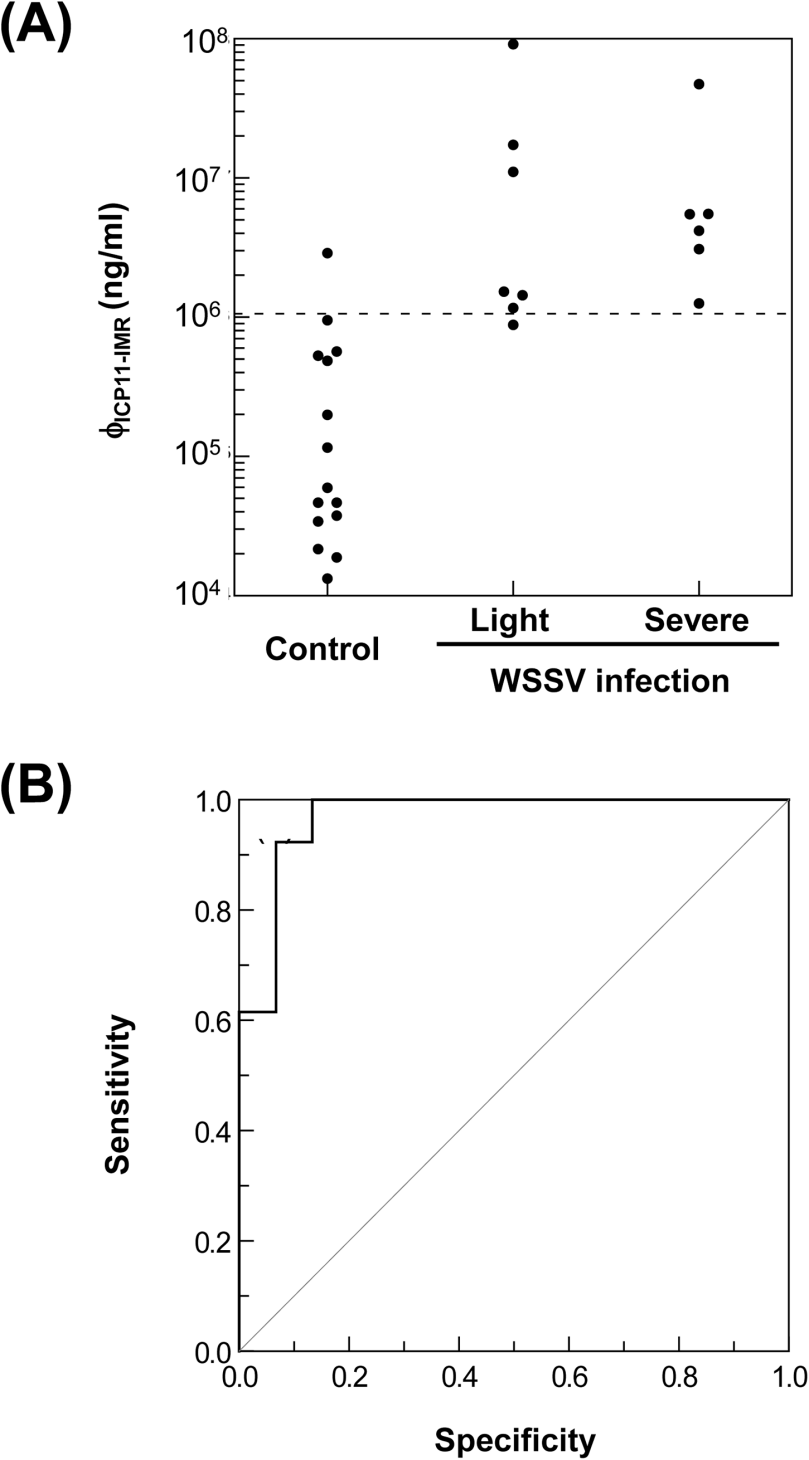
IMR detection of ICP11 protein in shrimp. (A) Measured ICP11 protein concentration (ϕ_ICP11-IMR_) in non-challenged shrimp (normal control) and in shrimp with a light or severe WSSV infection. The cut-off value (the dashed line) was based on ROC curve analysis of the IMR results. (B) Sensitivity and specificity of the IMR assay as determined by ROC curve analysis.

The IMR assay was used to detect the ICP11 concentrations (ϕ_ICP11-IMR_) in a total of 28 samples (Fig 7A). Detected ICP11 concentrations of samples collected from healthy shrimp (albeit with an apparent low-level persistent infection) were mostly < 1 x 10^6^ ng/ml, whereas the ϕ_ICP11-IMR_ of shrimp with a light or severe infection consistently exceeded 1 x 10^6^ ng/ml. Using receiver operating characteristic (ROC) curve analysis (Fig 7B), the cut-off value of ICP11 concentration between non-challenged shrimp with apparent persistent infection and WSSV-challenged shrimp with light/severe infection was 10.55 x 10^5^ ng/ml with a specificity of 0.933 and sensitivity of 0.923.

**Fig 8:**
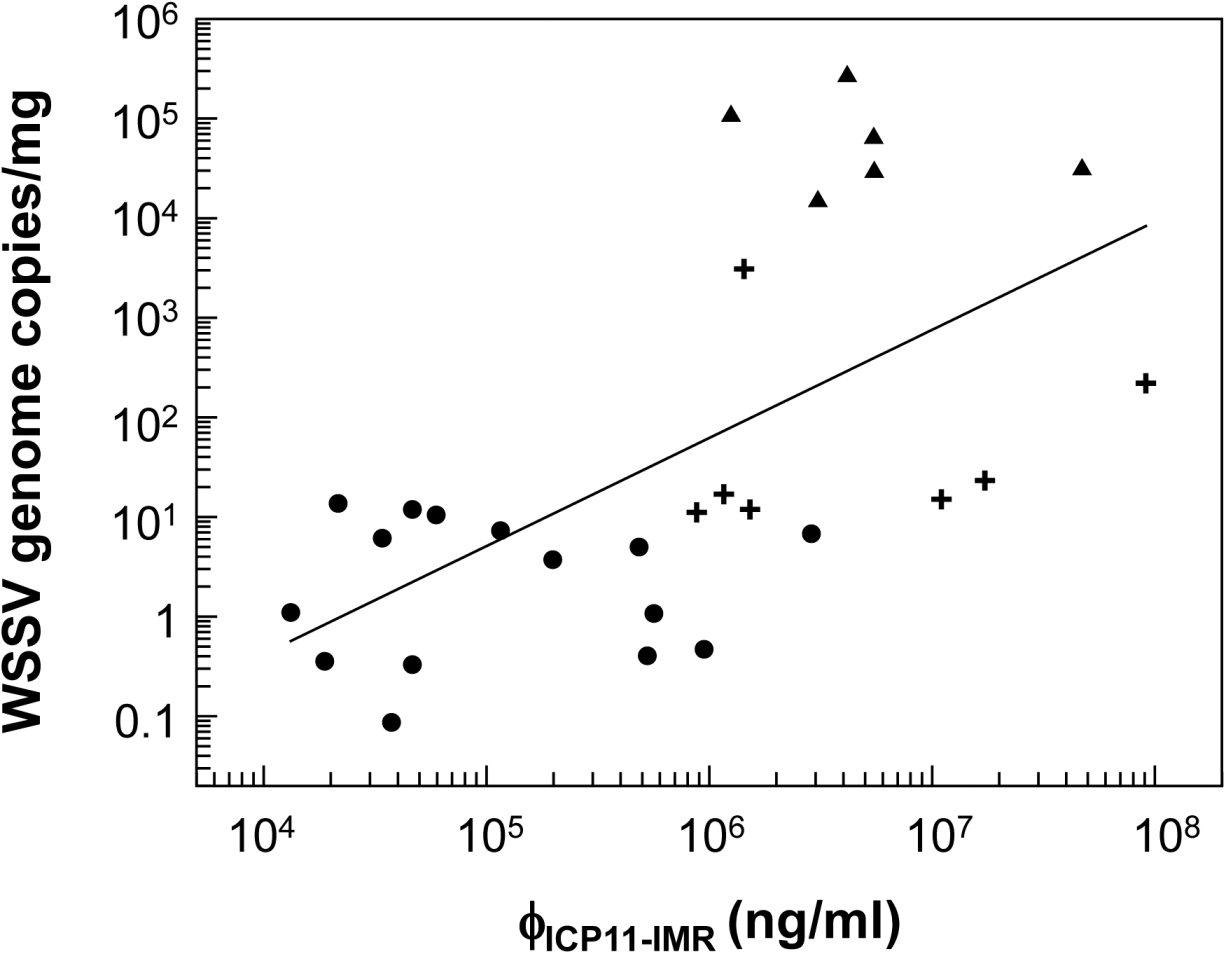
Correlation between detected ICP11-IMR concentration and WSSV copy number. Pleopod samples were collected from shrimp and subjected to IMR assay and real-time PCR. Dots indicate shrimp belonging to the non-challenged control group (0.08 ~ 7 WSSV copies/mg tissue); crosses indicate light infection (10 ~ 3100 WSSV copies/mg tissue); triangles indicate severe infection (1.57 x 10^4^ ~ 2.83 x 10^5^ WSSV copies/mg tissue).

Fig 8 plots the detected ICP11 concentrations (ϕ_ICP11-IMR_) against the number of WSSV genome copies per mg in shrimp in the control and WSSV-infected groups. We note that although the control shrimp had a low number of WSSV genome copies (< 10 copies / mg tissue; Fig 8) these same shrimp nevertheless had high ϕ_ICP11-IMR_ values of 10^4^~10^6^ ng/ml. Even though ICP11 is the most highly expressed WSSV protein, it seems unlikely that such a high concentration of the protein would be produced by so few copies of the virus; the ϕ_ICP11-IMR_ values may therefore not be truly representative of the actual quantity of ICP11 present in the lysates. The reason for this apparent discrepancy is not clear. The Western blots in Fig 2 eliminate the possibility that the polyclonal antibody may be directly cross-reacting with shrimp tissue, but unexpected non-specific cross-reaction with other proteins cannot be ruled out. Distorted ϕ_ICP11-IMR_ values might also arise if the ICP11 is in polymeric form and/or if it is bound to histone DNA (18) because these large molecules/complexes will cause a correspondingly large reduction in the χ_ac_ signal. Unfortunately, it may be that the ϕ_ICP11-IMR_ values calibrated by purified ICP11 cannot be directly applied to ICP11 in shrimp lysate. Clearly, more work will need to be done to investigate this issue.

## Conclusions

In this study, we developed an immunomagnetic reduction (IMR) assay to detect ICP11 (a highly expressed WSSV protein) in shrimp. To our knowledge, this protein has never been used as the target in an immune-based WSSV diagnostic system. A simple process was used to extract ICP11 protein from shrimp pleopods, and the protein was subsequently quantified using a high-sensitivity IMR assay. Despite some unexpectedly high ϕ_ICP11-IMR_ values in some of the putatively healthy shrimp, we nevertheless conclude that this ICP11-based IMR assay has great potential for the early detection of WSSV in shrimp.

## Supporting Information

S1 Table Detected IMR signals and their mean value, standard deviation (SD), and coefficient of variation (CV) for each ICP concentration in Fig 5. (DOCX)
