# Supplementary material for: A Novel Detection Platform for Shrimp White Spot Syndrome Virus Using an ICP11-Dependent Immunomagnetic Reduction (IMR) Assay
Source: PLoS One. 2015 Sep 18;10(9):e0138207. doi: 10.1371/journal.pone.0138207 (PMC4575139; doi:10.1371/journal.pone.0138207)
Supplement: S1 Table — (DOCX) [file pone.0138207.s001.docx]

**S1 Table. Detected IMR signals and their mean value, standard deviation (SD), and coefficient of variation (CV) for each ICP concentration in Fig. 5**

|  | **ϕICP11 (ng/ml)** | | | | | | | |
| --- | --- | --- | --- | --- | --- | --- | --- | --- |
|  | **0.1** | **1** | **10** | **10^2^** | **10^3^** | **10^4^** | **10^5^** | **10^6^** |
| 1st-measurement IMR (%) | 0.78 | 0.83 | 0.89 | 0.97 | 1.11 | 1.2 | 1.44 | 1.72 |
| 2nd-measurement IMR (%) | 0.75 | 0.82 | 0.91 | 1.01 | 1.08 | 1.19 | 1.46 | 1.74 |
| Mean value of duplicate IMR signals (%) | 0.765 | 0.825 | 0.9 | 0.99 | 1.095 | 1.195 | 1.45 | 1.73 |
| SD of duplicate IMR signals (%) | 0.021 | 0.007 | 0.014 | 0.028 | 0.021 | 0.007 | 0.014 | 0.014 |
| CV of duplicate IMR signals | 2.77% | 0.86% | 1.57% | 2.86% | 1.94% | 0.59% | 0.98% | 0.82% |
